# Altered Topological Properties of Static/Dynamic Functional Networks and Cognitive Function After Radiotherapy for Nasopharyngeal Carcinoma Using Resting-State fMRI

**DOI:** 10.3389/fnins.2021.690743

**Published:** 2021-07-14

**Authors:** Xi Leng, Chunhong Qin, Huan Lin, Mingrui Li, Kui Zhao, Hongzhuo Wang, Fuhong Duan, Jie An, Donglin Wu, Qihui Liu, Shijun Qiu

**Affiliations:** ^1^Medical Imaging Center, The First Affiliated Hospital of Guangzhou University of Chinese Medicine, Guangzhou, China; ^2^Department of Radiology, Guangdong Provincial People’s Hospital, Guangdong Academy of Medical Sciences, Guangzhou, China

**Keywords:** resting-state functional magnetic resonance imaging, graph theory, radiation induced brain injury, dynamic brain functional network, cognitive function, nasopharyngeal carcinoma

## Abstract

**Objectives:**

The purpose of this study was to (1) explore the changes in topological properties of static and dynamic brain functional networks after nasopharyngeal carcinoma (NPC) radiotherapy (RT) using rs-fMRI and graph theoretical analysis, (2) explore the correlation between cognitive function and changes in brain function, and (3) add to the understanding of the pathogenesis of radiation brain injury (RBI).

**Methods:**

Fifty-four patients were divided into 3 groups according to time after RT: PT1 (0–6 months); PT2 (>6 to ≤12 months); and PT3 (>12 months). 29 normal controls (NCs) were included. The subjects’ topological properties were evaluated by graph-theoretic network analysis, the functional connectivity of static functional networks was calculated using network-based statistics, and the dynamic functional network matrix was subjected to cluster analysis. Finally, correlation analyses were conducted to explore the relationship between the altered network parameters and cognitive function.

**Results:**

Assortativity, hierarchy, and network efficiency were significantly abnormal in the PT1 group compared with the NC or PT3 group. The small-world variance in the PT3 group was smaller than that in NCs. The Nodal ClustCoeff of Postcentral_R in the PT2 group was significantly smaller than that in PT3 and NC groups. Functional connectivities were significantly reduced in the patient groups. Most of the functional connectivities of the middle temporal gyrus (MTG) were shown to be significantly reduced in all three patient groups. Most of the functional connectivities of the insula showed significantly reduced in the PT1 and PT3 groups, and most of the functional connectivities in brain regions such as frontal and parietal lobes showed significantly reduced in the PT2 and PT3 groups. These abnormal functional connectivities were correlated with scores on multiple scales that primarily assessed memory, executive ability, and overall cognitive function. The frequency F of occurrence of various states in each subject differed significantly, and the interaction effect of group and state was significant.

**Conclusion:**

The disruption of static and dynamic functional network stability, reduced network efficiency and reduced functional connectivity may be potential biomarkers of RBI. Our findings may provide new insights into the pathogenesis of RBI from the perspective of functional networks.

## Introduction

Nasopharyngeal carcinoma (NPC) is a malignant tumor of the head and neck common in Asia, especially in southern China (Xia et al., 2017). Radiotherapy (RT) is the treatment of choice for NPC (Li et al., 2018), but RT may lead to the development of serious complications in the central nervous system, namely, radiation brain injury (RBI), which can be temporary and reversible or progressive and irreversible, with clinical manifestations ranging from mild fatigue to neurocognitive dysfunction and even death (Stone and DeAngelis, 2016). RBI not only significantly reduces the quality of life of patients but also imposes a great economic burden to families and society. Pertinently, we broadly divided RBI into three conventional periods: an acute response period (a few days to a few weeks), an early delayed period (1–6 months), and a late delayed period (>6 months) (Soussain et al., 2009). It has been found that RBI begins in the early delayed period and can persist into the late delayed period; also, functional abnormalities in the early delayed period can develop into late irreversible structural brain changes that cause permanent cognitive impairment (Attia et al., 2014). Therefore, there is an urgent need to detect early and potentially reversible brain functional impairment before severe irreversible structural damage and cognitive impairment occur in patients with NPC. Obtaining sensitive functional imaging markers of RBI not only helps to reveal the complex pathogenesis of RT-induced cognitive impairment but can also facilitate the early identification of RBI or even reverse its course.

Resting-state functional magnetic resonance (rs-fMRI) is simple, convenient, and reproducible; is widely used in the study of many neuropsychiatric diseases; and has also been progressively used in the study of brain function in RBI, which mostly focuses on brain function in local brain regions (Yang et al., 2019). However, the human brain is a highly complex and fine interconnected network with the ability to isolate and integrate information; brain diseases cannot only damage local brain regions but also lead to the reduction or disruption of connections within and between brain regions and abnormalities in the structure and function of brain networks. The graph theory-based complex brain network analysis method is a research tool that has emerged in recent years. Compared with previous analysis methods, it reveals the topological properties of brain networks from the perspective of information transfer, explains the internal working mechanism of brain networks, and analyzes MRI information more intuitively. These features enable us to understand how brain diseases affect cognitive function based on the basic properties of brain networks; elucidates the pathogenesis of brain diseases; and has been widely used in the study of central nervous system disorders such as Alzheimer’s disease, epilepsy, and schizophrenia (Ponten et al., 2007; He et al., 2008; Alexander-Bloch et al., 2013). The graph theory method provides a more systematic approach to the study of RBI, which has been less studied, and a previous graph theory-based DTI study found abnormalities in the topological properties of the whole-brain white matter structural network after RT for NPC (Chen Q. et al., 2020). This finding indicates that the processing patterns of the brain structural network have been altered in patients after RT, and compared to brain structural connectivity (SC), brain functional connectivity (FC) can more sensitively reflect changes in brain networks. We hypothesize that the topological properties of the functional brain networks of patients with NPC are also altered after RT, but their exact evolution is unknown. Identifying the altered topological properties of the whole-brain functional networks of patients will help us understand the neural mechanisms of RBI from the perspective of functional brain networks.

In contrast to static brain functional network analysis, dynamic functional network analysis, which has received increasing attention from researchers in recent years, can reflect the time-varying properties of the complex neural networks of the brain. During the dynamic process of neural activity, large-scale brain regions maintain temporary stable states in a highly modular form; these states transition from one kind of stable functional state to another with rapid and distinct transitions to each other, making the brain change from one kind of stable functional state to another kind of stable functional state. Dynamic network analysis can capture the time-varying properties on short time scales, reflect the changes and connections among the complex functional networks of the brain, and provide more information; this analysis has also been gradually used in the study of multiple brain functions and mechanisms of brain diseases (Du et al., 2018; Faghiri et al., 2018; Supekar et al., 2019), and dynamic functional network analysis can be mutually validated and complemented with static functional network analysis to jointly explore the neural mechanisms of brain diseases.

Cognitive decline is an important and sensitive indicator of damage to the central nervous system; exploring changes in cognitive function after RT in patients with NPC is important for early monitoring of RBI. Studies have found that patients with NPC have a variety of impaired cognitive functions after RT, but the MoCA scale, which is widely used in studies, is not sensitive enough to changes in some cognitive functions, such as executive function, verbal visual memory function, and attention (Chapman et al., 2012; Greene-Schloesser et al., 2012). Changes in cognitive function after RT for NPC need to be explored with a more comprehensive cognitive assessment scale. Therefore, in this study, we used rs-fMRI data and graph theory-based brain network analysis to explore the topological properties of static and dynamic functional networks in the whole brain after RT for NPC, to explore the pathogenesis of RBI, and to find more sensitive biomarkers for RBI diagnosis; we used a combination of several scales to further comprehensively explore the change pattern of cognitive function in NPC patients after RT and to elucidate its correlation with the evolution of brain function. To the best of our knowledge, this study is the first to explore the changes in topological properties of static and dynamic functional networks of the whole brain after RT for NPC using a graph-theoretic analysis and the first to investigate the correlation between cognitive function and changes in brain function using a combination of multiple scales.

## Materials and Methods

### Subjects

Fifty-four patients with pathologically confirmed NPC (41 males, 13 females; aged between 27 and 63 years; mean age, 45.69 years) and twenty-nine normal controls were included in this study. All patients underwent fractionated RT for the first time with three-dimensional conformal and intensity-modulated techniques (total dose/fraction dose/exposures, 66–74 Gy/1.8–2.0 Gy/30–35 times). Prior to MRI, we verified that patients did not exhibit intracranial tumors or intracranial invasion. Patients with hypertension, diabetes, heart disease, white matter degeneration, or vascular disease were excluded. Normal subjects constituted the control group, and the same exclusion criteria that were applied to RT subjects were applied to the control group (i.e., hypertension, diabetes mellitus, heart disease, white matter degeneration, and vascular disease). Traditionally, RT-induced neurological impairment can be described by the acute response period, early delayed period, and late delayed period, depending on the time of RT completion. Therefore, in our study, patients who had completed RT were divided into three groups according to the stage of RBI: patient group 1 (PT1) (0–6 months after RT, *n* = 16), patient group 2 (PT2) (>6 to ≤12 months after RT, *n* = 8), and patient group 3 (PT3) (>12 months after RT, *n* = 30). No statistically significant differences were found between the groups according to age, sex, or education. Demographic and clinical data are shown in Table 1. This study was approved by the Ethics Committee of First Affiliated Hospital of Guangzhou University of Chinese Medicine. The current study was carried out in accordance with the principles of the Declaration of Helsinki and the approved guidelines. All subjects signed informed consent before participating in the study.

**TABLE 1:** Demographics, clinical data, cognitive assessment of NPC patients after RT and NCs.

	**PT 1 group (*n* = 16)**	**PT 2 group (*n* = 8)**	**PT 3 group (*n* = 30)**	**NCs (*n* = 29)**	**Statistics**	***P*-value**
**Clinical characteristics**						
Age (years)	45.00 (32.25, 52.75)	46.00 (38.75, 57.75)	50.00 (40.25, 55.00)	37.00 (28.50, 52.00)	H = 5.438	0.142
Sex (M/F)	11/5	5/3	25/5	19/10	χ^2^ = 2.938	0.401
Education (years)	12.00 (12.00, 12.00)	10.50 (9.00, 15.75)	12.00 (9.00, 15.00)	15.00 (10.50, 16.00)	H = 7.532	0.057
**Cognitive scores**						
MoCA-B	25.00 (24.00, 26.00)	27.50 (25.25, 28.00)	26.00 (24.75, 27.00)	28.00 (27.00, 29.00)	H = 27.893	< 0.001*
DSST	46.00 (26.75, 57.00)	57.00 (40.25, 60.75)	41.00 (33.75, 43.50)	55.00 (40.00, 67.00)	H = 13.241	0.004*
DST forward	8.50 (6.00, 9.75)	9.50 (8.25, 11.75)	8.00 (7.00, 8.00)	9.00 (8.00, 9.50)	H = 14.027	0.003*
DST backward	4.00 (3.00, 5.00)	4.00 (4.00, 6.75)	4.00 (4.00, 5.00)	5.00 (5.00, 7.00)	H = 17.308	0.001*
DST	11.50 (9.00, 14.00)	15.00 (12.25, 16.75)	12.00 (10.75, 13.00)	14.00 (13.00, 15.50)	H = 18.843	< 0.001*
TMT-A	39.11 (27.45, 50.38)	40.78 (27.62, 47.94)	47.98 (37.29, 61.16)	30.91 (25.20, 40.87)	H = 15.772	0.001*
TMT-B	33.81 (24.09, 48.95)	39.54 (26.05, 48.48)	37.75 (30.03, 50.55)	28.71 (20.25, 35.82)	H = 11.180	0.011*
AVLT (immediate)	20.50 (17.25, 23.00)	20.50 (19.00, 21.00)	20.50 (17.75, 23.25)	25.00 (23.00, 29.50)	H = 21.088	< 0.001*
AVLT (5 min)	8.00 (7.00, 9.75)	9.00 (7.25, 10.75)	8.00 (6.75, 10.00)	10.00 (9.00, 11.00)	H = 12.907	0.005*
AVLT (20 min)	8.00 (7.00, 9.00)	8.50 (7.00, 10.00)	8.00 (6.00, 9.00)	10.00 (8.50, 11.00)	H = 18.152	< 0.001*
AVLT (recognition)	11.00 (10.00, 12.00)	12.00 (10.50, 12.00)	10.00 (9.00, 12.00)	12.00 (11.00, 12.00)	H = 12.330	0.006*

Data are presented as median [interquartile ranges (IQR)]. PT1, 0–6 months after RT; PT2, > 6 to ≤ 12 months after RT; PT3, > 12 months after RT; MoCA-B, montreal cognitive assessment-B; DSST, digit symbol substitution test; DST, digital span test; TMT, trail making test; AVLT, auditory verbal learning test; AVLT (immediate), auditory verbal learning test immediate recall; AVLT (5 min), auditory verbal learning test short-term delayed recall; AVLT (20 min), auditory verbal learning test long-term delayed recall. *P < 0.05, which was considered statistically significant.

### Neurocognitive Tests

All subjects underwent a series of neuropsychological tests, including the Montreal Cognitive Assessment-basic (MoCA-B), Auditory Verbal Learning Test (AVLT) (Zhao et al., 2015), Trail Making Test (TMT; including parts A and B) (Muir et al., 2015), Digit Symbol Substitution Test (DSST) (Chen X. et al., 2020), and Digital Span Test (DST, including forward and backward components) (Diamond, 2013). The AVLT consists of four parts: immediate recall, short-term delayed recall, long-term delayed recall, and recognition. It took subjects at least 35 min to complete the entire evaluation.

### Image Acquisition

All MRI data were acquired on a 3.0 T clinical scanner (SIGNA EXCITE; GE Healthcare, Chicago, IL, United States). We first acquired conventional T1-weighted, T2-weighted, and T2-FLAIR images as routine clinical MRI data to ensure that there were no visible lesions in the brain. Subsequently, rs-fMRI scans with echo-planar imaging sequences and high-resolution T1-weighted 3D image scans were performed sequentially. The imaging parameters were as follows: (1) rs-fMRI: repetition time (TR) = 2,100 ms, echo time (TE) = 35 ms, flip angle = 90°, acquisition matrix = 64 × 64, field of view = 240 × 240 mm2, axial images = 40, layer thickness = 3.8 mm, layer spacing = 0 mm, and voxel size = 3.75 × 3.75 × 3.8 mm3; (2) 3D-T1WI: TR = 5.5 ms, *TE* = 1.5 ms, flip angle = 12°, acquisition matrix = 256 × 256, field of view = 256 × 256 mm2, sagittal slices = 166, no interslice gap, and voxel size = 1 × 1 × 1 mm3. During the rs-fMRI scan, eachsubject was instructed to close his eyes and try to remain still, not to think systematically about anything, and not to fall asleep.

### Data Preprocessing

The raw images in DICOM format were converted to NIFTI format using dcm2nii software, and then the data were preprocessed using Data Processing Assistant for rs-fMRI (DPARSF) (Chao-Gan and Yu-Feng, 2010) and Statistical Parametric Mapping (SPM12)^1^ on the MATLAB 2013b (MathWorks, Natick, MA, United States) platform. The first 5 volumes of each subject were discarded to exclude the instability of the initial MRI signal, and the 235 remaining volumes were slice-time corrected and three-dimensional head motion corrected. Because the scanned images were interleaved scan in the plus direction starting at odd, the slice order was [1:2:39 2:2:40] and the reference slice was 39. The subjects whose head motion was more than 3 mm in either the x, y, or z direction or was more than 3 of any angular motion were rejected (Liu et al., 2015). Moreover, the head motion profiles were matched among the three PT groups and NCs (*P* > 0.05 in mean FD and in any direction). After that, the individual high-resolution T1WI images were linearly registered to the realigned mean functional images, subsequently, the co-registered images were spatially normalized to Montreal Neurological Institute (MNI) 152 template and resampled to 3 × 3 × 3 mm^3^. No smoothing was performed in order to avoid introducing artificial local spatial correlation for connectomic (Zuo et al., 2012; Wang et al., 2013; Soares et al., 2016). Then, the data were linearly detrended and temporally filtered using a 0.01∼0.08 Hz band. Finally, nuisance covariates consisting of 24 motion parameters (Friston et al., 1996), white matter (WM) and cerebrospinal fluid (CSF) were regressed out, except for the global mean signal (Saad et al., 2012).

### Static Functional Network Construction and Network Analysis

#### Network Construction

The GRETNA toolkit^2^ was used for the binarized static network construction and graph-theoretic analysis of the whole-brain network. Automated anatomical labeling (AAL) was used to divide the whole brain into 116 regions of interest, which were defined as the brain nodes of the network. The mean time series of the 116 brain regions of interest were extracted for each subject separately, and the Pearson correlation coefficients between each pair of regions were calculated, which were defined as the edges of the network. Then, a 116 × 116 Pearson’s correlation matrix for each subject was created. To improve normality, Fisher’s r-to-z transformation was performed. According to a predefined threshold on the Pearson’s correlation coefficient, if the Pearson’s correlation coefficient was greater than the threshold between each pair of regions, it was counted as one link. Thus, the Pearson’s correlation matrix was converted into a binarized matrix.

#### Network Analysis

About the choose of sparsity value, there was no available criteria for which sparsity value was the most biologically meaningful. In order to possess small-world properties and to ensure that sigma (σ) was greater than 1.1, the study applied few sparsity thresholds ranging from 0.05 to 0.4 with an interval of 0.01 (Zhang et al., 2011) to calculate the whole-brain functional network topological properties. Both global and local network measures were investigated. The global network measures included (1) Assortativity, it describes the correlation between the degrees of pairs of connected nodes, (2) Hierarchy, it is used to identify the presence of a hierarchical organization in a network, (3) Network efficiency, it characterizes the efficiency of parallel information transfer of that node in the network, (4) Small-world, it is the network that combines high clustering factor and shortest path length and (5) Synchronization, it measures how likely it is that all nodes fluctuate in the same wave pattern; the local network measures included (1) Betweenness Centrality, it defines the centrality of a node from the perspective of information flow, (2) Degree Centrality, it portrays the degree of centrality of a node in the network in terms of its degree, (3) Nodal ClustCoeff, it measures the degree of grouping of the nodal in network, (4) Nodal Efficiency, it characterizes the efficiency of parallel information transfer of that node in the network, (5) Nodal Local Efficiency, it measures how efficient the communication is among the first neighbors of this node when it is removed and (6) Nodal Shortest Path Length, it portrays the optimal path for information from a node in the network to reach another node.

### Dynamic Functional Network Construction, Network Analysis and Clustering Analysis

#### Network Construction

SPM12 and DynamicBC (Liao et al., 2014) were applied to construct the dynamic whole-brain functional network using the sliding window technique with the AAL116 template. In this study, the window size was 30 TR, and the overlap was 0.9. These parameters were chosen so that each subject had approximately 69 time points, and each time point corresponded to a 116 × 116 network matrix. The final dynamic functional network matrix dimension of each subject was 69 × 116 × 116.

#### Network Analysis

The same network topological properties were investigated in the dynamic whole-brain network as were examined in the static whole-brain functional network using the same sparsity and interval in GRETNA. However, the variance between the 69 time points of each global and local measure was also calculated.

#### Clustering Analysis

The dynamic functional network matrix of each subject was subjected to cluster analysis. Clustering was based on city block analysis and the commonly used dynamic network measures were calculated after the completion of the cluster analysis, including F (frequency of occurrence of various states for each subject), MDT (mean dwell time of each state in TR), NT (number of state transfers), and TM (state transfer matrix probability of conversion of one state to other states).

### Statistical Analysis

SPSS 20.0 (SPSS, Inc., Chicago, IL) was used to evaluate the differences of clinic data among study groups. Normal distributions were tested using the Shapiro Wilk test. Beacese the age, education and cognitive scale scores were basically not normally distributed, then the nonparametric Kruskal Wallis test was used to compare the inter-group differences. *P* < 0.05 was considered statistically significant.

GRETNA was used to compared the differences of global and local network measures and dynamic network cluster analysis measures among the four groups using a one-way 4-level group analysis. When comparing the differences in the network topological properties among the 4 groups, the area under the curve (AUC) of each network measure was used in the static functional network, whereas, the variance between the 69 time points of each network measure was used in the dynamic functional network. Sex, age, and education were used as covariates in both graph theoretical network analysis and clustering analysis, with Bonferroni correction (<0.05) for the global measure, F, and MDT and multiple comparison false discovery rate (FDR) correction (*P* < 0.05) for the local measure, NT and TM. Static whole-brain networks were analyzed to identify significantly different functional connections using network-based statistics (NBS) (Zalesky et al., 2012) with sex, age, and education as covariates (*P* < 0.001) using a one-way, 4-level group analysis and a nonparametric permutation method (10,000 permutations, *P* < 0.05) to determine whether these functional connections were significantly altered.

For the correlation analysis, the generalized linear model (GLM) model was firstly used to regress the effects of covariates (age, gender and education), then the Spearman correlation coefficients between the network topology attribute index, network topology attribute index variance and static functional connectivity displaying intergroup differences and MoCA-B, AVLT, TMT, DST, and DSST scores in the post-RT group were calculated. Moreover, Bonferroni corrections were used for multiple comparisons. *P* < 0.05 was considered statistically significant.

## Results

### Global Parameters

**FIGURE 1:**
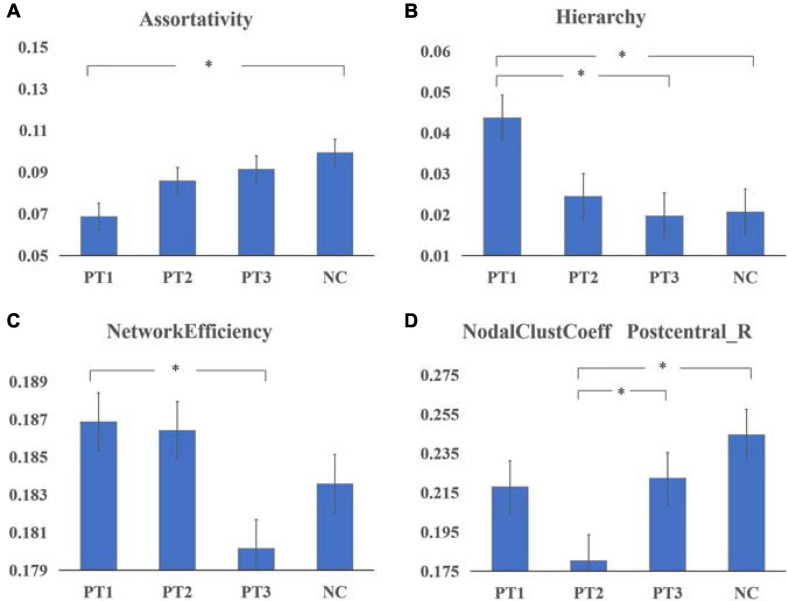
Global parametders **(A–C)** and nodal parameters **(D)** of the static brain functional network in patients with NPC in the 0–6 months post-RT (PT1) group, > 6 to ≤ 12 months post-RT (PT2) group, and > 12 months post-RT (PT3) group vs. normal controls (NCs). **(A)** Assortativity was significantly smaller in the PT1 group than in the NC group (*P* = 0.011). **(B)** Hierarchy values were significantly larger in the PT1 group than in the PT3 and NC groups (*P* = 0.048 and *P* = 0.039). **(C)** Network efficiency was significantly greater in the PT1 group than in the PT3 group (*P* = 0.003). **(D)** The nodal clustering coefficient of Postcentral_R in the PT2 group was significantly smaller than that in the PT3 and NC groups (*P* = 0.023 and *P* = 0.000). *Means that the difference is statistically significant after multiple comparisons.

**FIGURE 2:**
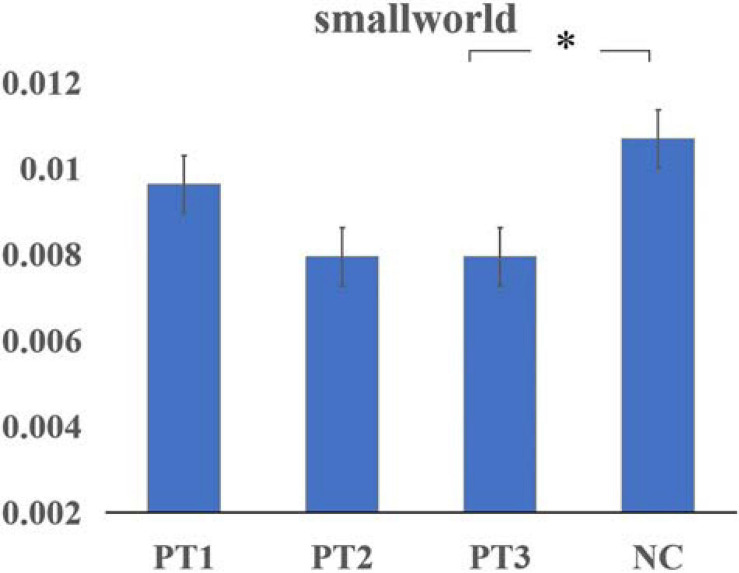
The small-world variance of the dynamic functional network in the PT3 group was significantly smaller than that of the NC group (*P* = 0.027). *Means that the difference is statistically significant after multiple comparisons.

Assortativity was significantly smaller in the PT1 group than in the NC group (*P* = 0.011). Hierarchy values were significantly larger in the PT1 group than in the PT3 and NC groups (*P* = 0.048 and *P* = 0.039). Network efficiency was significantly greater in the PT1 group than in the PT3 group (*P* = 0.003; Figure 1). Small-world differences between the groups were initially significant but were not significant in *post hoc* comparisons. The small-world variance of the PT3 group was significantly smaller than that of the NC group (*P* = 0.027; Figure 2). The difference between groups for Synchronization was not significant (*P* = 0.120). However, when multiple comparisons were corrected, no significant correlation between above global parameters and neurocognitive tests was found.

### Nodal Parameters

The nodal clustering coefficient of Postcentral_R in the PT2 group was significantly smaller than that in the PT3 (*P* = 0.023) and NC groups (*P* = 0.000) (Figure 1). And the differences between groups for betweenness centrality, degree centrality, nodal efficiency, nodal local efficiency, nodal shortest path length were not significant.

### Functional Connectivity

**FIGURE 3:**
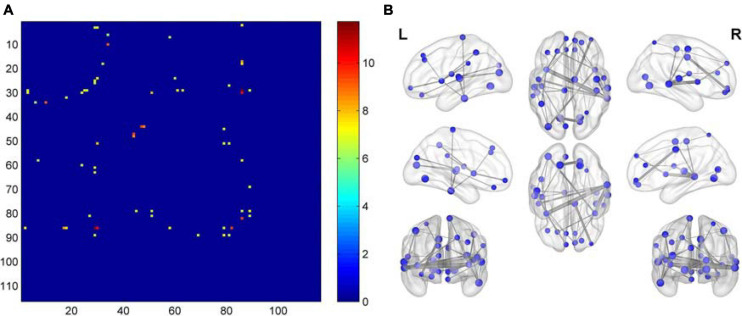
Matrix plots and brain maps of significant *F*-values for differences in functional connectivity. **(A)** The dots represent *F*-values with differences, the darker the color, the more significant the difference. **(B)** On the brain map, the lines between brain regions represent differential *F*-values, and the thicker the line, the more significant the difference.

Compared with the NC group, the functional connectivities of the post-RT groups differed significantly across time, with the vast majority of the post- RT patient groups having significantly reduced functional connectivities. Reduced functional connectivities were mostly located in the temporal lobe, insula, frontal lobe, and parietal lobe (Figure 3 and Table 2); most of the functional connectivities of the middle temporal gyrus (MTG) were shown to be significantly reduced in all three patient groups after RT than in the control group. Most of the functional connectivities of the insula (INS) showed significantly reduced in the PT1 and PT3 groups than in the control group, and most of the functional connectivities in brain regions such as frontal and parietal lobes showed significantly reduced in the PT2 and PT3 groups than in the control group. These abnormal functional connectivities were correlated with scores on multiple scales, such as the MoCA-B, AVLT, TMT, DSST, and DST which primarily assessed memory, executive ability, and overall cognitive function (Table 3). However, when multiple comparisons were corrected, no significant correlation between above functional connectivities and neurocognitive tests was found.

**TABLE 2:** Regions with altered functional connectivity in NPC patient groups after RT by NBS analysis.

**Between-group differences in functional connectivity**	**The name of the connecting edge**
The PT1, PT2 and PT3 groups were significantly reduced than the NC group	edge SFGdor.L_INS.R, edge PreCG.R_MTG.R, edge ROL.L_MTG.R, edge ROL.R_MTG.R, edge INS.L_MTG.R, edge INS.R_MTG.R, edge PoCG.R_MTG.R, edge HES.L_MTG.R
The PT1 and PT3 groups were significantly reduced than the NC group	edge SFGdor.L_INS.L, edge ORBsupmed.L_INS.L, edge ORBsupmed.R_INS.L, edge SFGmed.R_INS.R, edge MFG.L_PoCG.R, edge REC.L_STG.L
The PT2 and PT3 groups were significantly reduced than the NC group	edge ROL.R_ACG.R, edge ORBsup.R_DCG.R, edge ORBmid.R_DCG.R, edge CAL.R_LING.L, edge CAL.R_LING.R, edge INS.R_MOG.L, edge INS.L_SMG.L, edge CUN.L_HES.L, edge MOG.L_HES.L, edge MOG.L_STG.L, edge STG.R_MTG.R, edge INS.L_ITG.L, edge HES.L_ITG.L, edge STG.L_ITG.L
The PT2 group was significantly reduced than the NC group	edge ROL.R_ACG.R
The PT3 group was significantly reduced than the NC group	edge SFGmed.R_SPG.R
The PT3 group was significantly reduced than the PT1 and NC groups	edge PCL.L_ITG.L

*PT1, 0–6 months after RT; PT2, > 6 to ≤ 12 months after RT; PT3, > 12 months after RT; NC, normal control; SFGdor.L, Frontal_Sup_L; INS.R, Insula_R; PreCG.R, Precentral_R; MTG.R, Temporal_Mid_R; ROL.L, Rolandic_Oper_L; ROL.R, Rolandic_Oper_R; INS.L, Insula_L; PoCG.R, Postcentral_R; HES.L, Heschl_L; ORBsupmed.L, Frontal_Mid_Orb_L; ORBsupmed.R, Frontal_Mid_Orb_R; SFGmed.R, Frontal_Sup_Medial_R; MFG.L, Frontal_Mid_L; REC.L, Rectus_L; STG.L, Temporal_Sup_L; ACG.R, Cingulum_Ant_R; ORBsup.R, Frontal_Sup_Orb_R; DCG.R, Cingulum_Mid_R; ORBmid.R, Frontal_Mid_Orb_R; CAL.R, Calcarine_R; LING.L, Lingual_L; LING.R, Lingual_R; MOG.L, Occipital_Mid_L; SMG.L, SupraMarginal_L; CUN.L, Cuneus_L; STG.R, Temporal_Sup_R; ITG.L, Temporal_Inf_L; SPG.R, Parietal_Sup_R; PCL.L, Paracentral_Lobule_L.*

**TABLE 3:** Correlations between reduced functional connectivity and cognitive function in NPC patients (before multiple comparisons).

**Group**	**Functional connectivity**	**Cognitive tests**	**Correlation coefficient(r)**	***P*-valve**
PT1	ORBsupmed.L-INS.L	MoCA-B	0.762	0.001
	ORBsupmed.L-INS.L	TMT-A	−0.694	0.003
	SFGdor.L-INS.R	DSST	0.556	0.025
	SFGdor.L-INS.R	DST backward	0.570	0.021
	ORBmid.R-DCG.R	MoCA-B	0.520	0.039
	ORBmid.R-DCG.R	DSST	0.582	0.018
	ROL.R-MTG.R	DSST	0.543	0.030
	ROL.R-MTG.R	DST backward	0.544	0.030
PT2	CAL.R-LING.R	AVLT recognition	0.714	0.047
	MOG.L-HES.L	AVLT recognition	0.714	0.047
	ORBmid.R-DCG.R	DST	0.786	0.021
	STG.R-MTG.R	DST	0.738	0.037
	INS.L-ITG.L	DST	0.762	0.028
	PCL.L-ITG.L	TMT-B	−0.749	0.033
PT3	ORBmid.R-DCG.R	DST backward	0.489	0.006
	CUN.L-HES.L	DST backward	0.378	0.039
	ROL.L-MTG.R	DST backward	0.394	0.031
	ROL.L-MTG.R	DST forward	0.462	0.010
	PoCG.R-MTG.R	DST forward	0.469	0.009
	PoCG.R-MTG.R	DST backward	0.555	0.001
	PoCG.R-MTG.R	DSST	0.415	0.023
	INS.R-MTG.R	DSST	0.370	0.044
	STG.R-MTG.R	DSST	0.439	0.015
	STG.L-ITG.L	DST	0.374	0.042

*Reduced functional connectivity was positively correlated with MoCA-B, DST, AVLT, and DSST scores and were negatively correlated with TMT scores before multiple comparisons, after Bonferroni corrections, no significant correlation was left. ORBsupmed.L, Frontal_Mid_Orb_L; INS.L, Insula_L; INS.R, Insula_R; SFGdor.L, Frontal_Sup_L; MTG.R, Temporal_Mid_R; ORBmid.R, Frontal_Mid_Orb_R; CAL.R, Calcarine_R; DCG.R, Cingulum_Mid_R; ROL.R, Rolandic_Oper_R; ROL.L, Rolandic_Oper_L; LING.R, Lingual_R; MOG.L, Occipital_Mid_L; HES.L, Heschl_L; STG.R, Temporal_Sup_R; ITG.L, Temporal_Inf_L; PCL.L, Paracentral_Lobule_L; CUN.L, Cuneus_L; PoCG.R, Postcentral_R.*

### Dynamic Network Clustering Analysis

**FIGURE 4:**
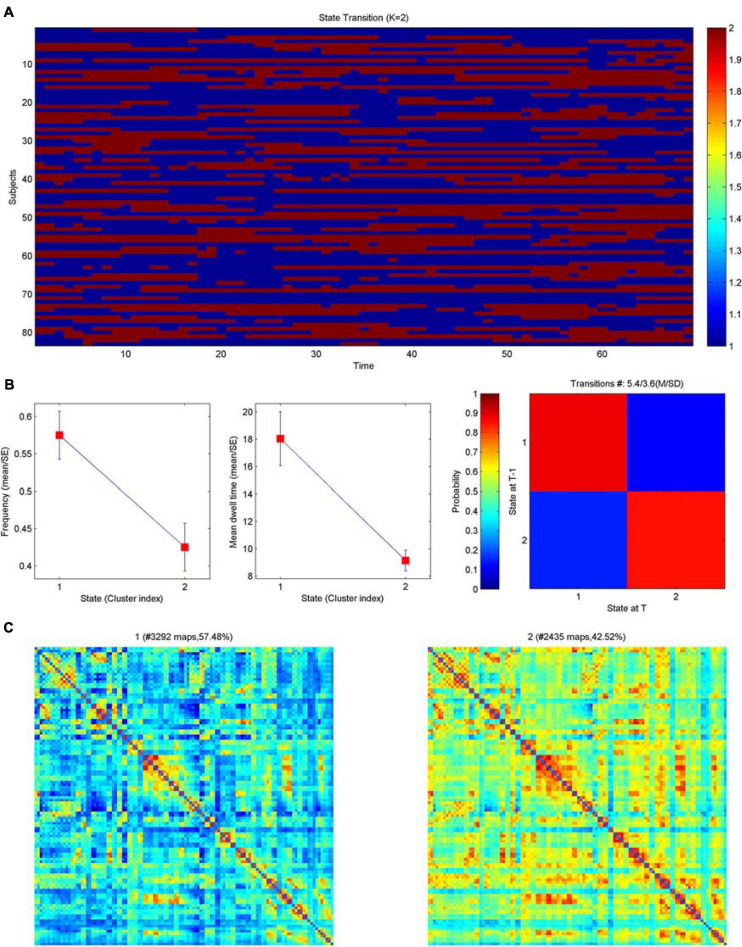
Clustering analysis of dynamic brain functional networks. **(A)** Brain network distribution of each subject over time. Blue represents state 1; red represents state 2; the horizontal axis is time, which can also be interpreted as the number of networks (69) and the vertical axis is the subject. **(B)** Left: average frequency of the two network states (corresponding to metric F below), middle: average dwell time of the two network states (corresponding to the metric MDT below), right: state transfer moment; blue represents state 1 and red represents state 2. **(C)** The matrix plot distribution of the two networks is shown on the left for state 1 with 57.48% and on the right for state 2 with 42.42%, both of which together are 100%.

The frequency F of occurrence of various states in each subject differed significantly, and the interaction effect of group and state was significant (*F* = 2.999, *P* = 0.036). At the group level, the probability of occurrence of state 1 in the PT2 group was significantly greater than that of state 2. At the state level, the probability of the PT2 group being in state 1 was significantly greater than that of the PT3 group, and the probability of the PT2 group being in state 2 was significantly less than that of the PT3 group (Figure 4). And the differences between groups for MDT (mean dwell time of each state in TR), NT (number of state transfers), and TM (state transfer matrix probability of conversion of one state to other states) were not significant.

## Discussion

This study was the first to use a graph-theoretic analysis to investigate the changes in the topological properties of resting-state static and dynamic functional networks in patients with NPC with normal, conventional cranial MRI performance at different times after RT and was the first to use a combination of multiple cognitive scales to analyze the correlation between changes in functional networks and cognitive impairment. We obtained the following results: (1) The PT1 group showed significant abnormalities in global properties of the static functional network (including assortativity, hierarchy, network efficiency) after radiotherapy; (2) The nodal clustering coefficient of Postcentral_R in the PT2 group was significantly smaller than that in the PT3 and NC groups; (3) The post-radiotherapy group had significantly reduced brain functional connectivities compared to normal controls; (4) The small-world variance of the dynamic functional network was significantly reduced in the PT3 group after radiotherapy, and the brain network exhibited inertia. Meanwhile in dynamic function networks, the frequency F of occurrence of various states in each subject differed significantly, and the interaction effect of group and state was significant.

### Global Parameters

A previous graph theory-based DTI study (Chen Q. et al., 2020) found significantly reduced global properties of structural networks in patients in the early delayed period after RT for NPC and suggested that RT may have affected the information transfer in early whole-brain neurostructural networks. Our study found significant abnormalities in global properties of functional networks after RT, including assortativity, hierarchy, network efficiency, small-world in the static functional network and small-world variance in the dynamic functional network. The 0–6 month post-RT group (PT1 group) had significantly smaller assortativity values than the NC group. Assortativity is used to examine whether nodes with similar degree values tend to connect to each other and describes the correlation between the degrees of connected node pairs (Deuker et al., 2009). Larger assortativity values represent more efficient network information transfer and indicate that the network is more robust (Newman, 2002). The assortativity of the functional network was significantly reduced in the early delayed period after RT, indicating that the efficiency of information transfer and the robustness of the network were reduced at this time. Hierarchy is a basic characteristic of brain networks, and higher values of hierarchy represent increased clustering of the network; however, higher hierarchy is associated with higher modularity, whereas, the connectivity of some nodes between different modules decreases (Ravasz and Barabási, 2003). The hierarchy values of the PT1 group were significantly greater than those of the PT3 and NC groups, implying that during the early delay period, the functional brain network undergoes reorganization as a reconstructive mechanism to maintain the overall brain function, resulting in increased clustering of the brain network; at the same time, this reorganization may also lead to a decrease in the efficiency of some nodes. In addition, the network efficiency values in the PT1 group were significantly greater than those in the PT3 group, also suggesting that a certain reconstructive effect may occur in the brain network early after RT, a reconstructive effect that was also confirmed in a previous study (Ding et al., 2018); this effect ensures that the functional brain network maintains a certain efficiency of information transfer and thus normal cognitive function. Abnormal changes in global properties may represent radiation-induced brain damage, and our findings suggest that RBI is already present in the early delayed period, with some properties significantly reduced and some properties showing some degree of compensation.

Small-world measurements showed a tendency to be abnormal in both static and dynamic functional networks, indicating some degree of abnormality in the efficiency of the brain network after RT. The post hoc comparison of small-world measurements in the static network was not significant, but in the dynamic network, small-world variance had significant abnormalities. Compared with the NC group, the variance of small-world properties of dynamic brain networks in the post-RT groups all showed a decreasing trend, which was significant in the PT3 group. This means that the variance in small-world properties over time was significantly smaller in the PT3 group than in the NC group. It was also reduced in the PT2 group, but due to the small number of people in the PT2 group, statistically significant differences were not reached. We believe that the small-world variance of the dynamic brain network after RT appeared inert, especially in the late delayed period. The small-world network property refers to the efficient and low energy-consuming network property of a network with a high clustering coefficient and a small shortest path length, which ensures efficient information separation and integration while simultaneously maximizing efficiency and minimizing energy consumption Many diseases exhibit abnormal small-world properties and are even considered to be caused by disorders of small-world properties, such as Alzheimer’s disease and schizophrenia (Mallio et al., 2015), which are characterized by changes in network efficiency and clustering (Rubinov et al., 2009). The results of static and dynamic functional networks in this study corroborate each other, indicating that the small-world property is an indicator of brain function in RBI, and small-world brain networks appear significantly inert in patients in the late delayed phase after RT. The efficiency of the brain functional network weakened, energy consumption increased, and the stability and balance of the brain functional network were significantly impaired. In addition, we hypothesized that the dynamic network in the PT2 group would also show more significant inertia, which needs to be explored by increasing the sample size in the next study. We introduced the topological properties of dynamic functional networks into the study of RBI for the first time, which could complement the topological properties of static functional networks, suggesting that small-world networks deserve close attention. The findings of global parameters in our study suggested that changes in the overall parameters of static and dynamic brain functional networks may be used as potential biomarkers of RBI.

### Nodal Parameters

Nodal parameters can detect the activity, importance and influence of a region in network communication. Node 58 (Postcentral_R) of the PT2 group had a significantly smaller nodal clustering coefficient than the PT3 and NC groups. In graph theory, the clustering coefficient is a measure of the degree of node clustering; the higher the clustering coefficient, the more connected to the surrounding nodes, and the higher the clustering coefficient, the closer the connection with surrounding nodes and the stronger the information dissemination ability. Previous studies found that white matter structural networks of the parietal lobe would be damaged after RT for NPC, and Chen Q. et al. (2020) used graph theory to find significant abnormalities in the node parameters of the parietal lobe. Based on the principle that structure determines function, it can be assumed that the functional node parameters of the parietal lobe would also be altered; our study obtained similar results. A functional study (Ding et al., 2018) using the posterior cingulate cortex (PCC) as a seed point found a significant reduction in functional connectivity in several parietal regions of the left inferior parietal lobule and the default mode network (DMN) in the 6–12 month group; this reduction in connectivity recovered after 12 months, and the investigators concluded that the most severe impact on local functional activity after RT occurred at 6–12 months, which is consistent with our findings. We speculated that changes in the early phase may be due to reconstructive effects, and the nodal clustering coefficient was not significantly reduced. The most severe damage occurred at 6–12 months, when the local brain network information dissemination capacity was significantly reduced, after which it may gradually recover. This result suggests that brain functional impairment after RT for NPC is not only limited to the temporal lobe but also occurs in parietal lobe, perhaps due to abnormalities in functional connectivity.

### Functional Connectivity

Our study found that a total of 31 brain functional connectivities were significantly reduced in the post-RT groups compared with the NC group, and these abnormal functional connectivities were located in multiple brain regions, mainly the temporal lobe, insula, frontal lobe, and parietal lobe, whereas, multiple cognitive function scores were significantly abnormal, and their functional connectivities were correlated with multiple cognitive function scores. Among these connections, the largest number of functional connectivities occurred in the temporal lobe; there were 16 connections, including functional those within the temporal lobe and between the temporal lobe and other brain regions. Compared to NCs, functional connectivities in the temporal lobe such as the MTG were significantly reduced in all three post-RT groups, suggesting that damage to the temporal lobe is more fixed in patients with nasopharyngeal carcinoma after RT, which is consistent with the results of previous studies. Temporal lobe is closest to the radiation field of RT for NPC and is at risk of injury when the radiation dose received exceeds the tolerance range of brain tissue (Xiong et al., 2013), whereas, the temporal lobe belongs to the DMN, and the functional connectivities within the DMN are very sensitive to radiation (Ding et al., 2018). Cognitive function-related studies have shown that diminished functional connectivity in the DMN is associated with memory, attention, and executive function impairment (Sestieri et al., 2011); similar results were found in this study, functional connectivity of MTG, superior temporal gyrus (STG) and inferior temporal gyrus (ITG) in three patient groups after RT was positively correlated with DSST, DST, DST-FORWARD and DST-BACKWARD scores, and negatively correlated with TMT-B scores. No significant correlation was found after multiple comparison correction. This may be partly due to the relatively rigorous calculations. Nevertheless, we believe our study is still relevant in providing direction for future research in this field. Previous studies have also concluded that even though there was no significant correlation after correction for multiple comparisons, it is still relevant to uncover the pathogenesis of cognitive impairment (Xia et al., 2013). Radiation may impair cognitive function, memory, and executive function by damaging functional connectivity in the temporal lobe of the brain. Previous RBI studies have shown that temporal brain function is related to memory and executive function, but the MoCA scale was more frequently used and was not sensitive enough (Qiu et al., 2018). We used a combination of multiple cognitive scales for the first time to uncover the correlation between functional connectivity and cognition in a more sensitive way. In addition, we found that most of the functional connectivities involving the left Heschl gyrus (HES.L), bilateral superior temporal gyrus (STG.L and STG.R), and left inferior temporal gyrus (ITG.L) were significantly reduced in the PT2 and PT3 groups than in the NC group; also, most of the PT1 group did not show significantly reduced functional connectivities, whereas, the functional connectivities between the ITG.L and the left paracentral lobule (PCL.L) appeared to be significantly reduced in the PT3 group than in the PT1 group. Previous studies found that the functional and structural connectivities of the inferior temporal gyrus were often compensatorily increased in the acute early period after RT (Chen Q. et al., 2020), and early temporal lobe increases in local brain functional activity can predict severe late temporal lobe necrosis (Ding et al., 2018). We speculate that the functional connectivity of the HES.L, STG.L, STG.R, ITG.L in this study may also have undergone reconstructive increases early on and resulted in the above findings; also, over time, the reconstruction diminished, and functional connectivity became significantly impaired in the PT2 and PT3 groups.

In studies of RBI (Yang et al., 2019; Chen Q. et al., 2020), the insula has been shown to be a brain region that is very sensitive to radiation and prone to RBI. Also, studies (Karunamuni et al., 2016) have found that cortical atrophy after cranial RT is most pronounced in the temporal lobe and limbic system, of which the insula is a part, which may explain why the insula was the second most severely functionally damaged brain region after the temporal lobe in the present study. It has also been suggested that radiation damage to the insula could be a secondary injury related to radiation damage to the temporal lobe, where radiation indirectly damages the insula by damaging the functional connectivity between the insula and temporal lobe (Qiu et al., 2018). Our study found 11 functional connectivities between the insula and the middle temporal gyrus (MTG.R) and ITG.L that were significantly reduced after RT; were positively correlated with MoCA-B, DSST, and DST-BACKWARD scores; and were negatively correlated with TMT-A scores in early delayed period. These findings suggest that radiation may impair the functional connectivities between the sensitive insula and other brain regions through direct damage or indirect damage, thus affecting the patients’ overall cognitive function, executive function and memory, and the insula has also been shown to play a key role in higher cognitive control and attentional processes in previous studies (Menon and Uddin, 2010). In addition, most of the functional connectivity of the insula in the present study showed significantly reduced in the PT1 and PT3 groups than in the control group, implying that the damage to the insula was more pronounced both early and late after radiotherapy, and the damage was not significantly repaired with the accumulation of time, which is similar to the findings of previous studies, where the PT2 group perhaps showed reconstruction of functional connectivity without being significant. Previous studies have shown that radiation damage to the insula has a cumulative effect that worsens with increasing radiation dose and with a “decrease without recovery” pattern of insula ReHo (Yang et al., 2019). Our study and previous findings implied that brain function in the insula is prone to radiation damage and persistently worsens; therefore, more attention and protection of this vulnerable insula is needed in future RT.

Most of the functional connectivities in brain regions such as frontal and parietal lobes showed significantly reduced in the PT2 and PT3 groups than in the control group, implying a possible reconstruction of functional connectivity in the PT1 group. It was shown that structural connectivity of the inferior frontal gyrus in the early delayed period was significantly reduced, whereas, structural connectivity of the superior frontal gyrus in the late delayed period was reduced after RT (Chen Q. et al., 2020). Our study showed that functional connectivity in the frontal lobe was also significantly impaired after RT, with multiple functional connectivities significantly reduced and positively correlated with MoCA-B, DSST, DST-BACKWARD, and DST scores in three patient groups. The frontal lobe is a constituent region of the DMN and central execution network (CEN) and is involved in higher-order cognitive processes; frontal lobe functional impairment may lead to cognitive deficits, especially in working memory and executive functions (Astle et al., 2015). Our study showed that frontal lobe functional impairment was associated with reduced memory and executive function after RT. Ding (Ding et al., 2018) found that fALFF in the superior frontal area was significantly elevated in the 0–6 months group after RT. Our study found similar results: part of the functional connectivity of the superior frontal area in the PT1 group did not show significant diminution, probably due to reconstruction. The frontal lobe was outside the irradiated field, and the study speculated that this may be plasticity on temporal lobe dysfunction and that this reconstruction may contribute to the maintenance of normal cognitive function (Levin et al., 2009). We also found that the functional connectivity of the postcentral gyrus (PoCG.R) and superior parietal lobule (SPG.L) to the frontal lobe was also significantly reduced after RT. The parietal lobe was also shown to be susceptible to radiation brain damage, with significant abnormalities in structural connectivity, gray matter volume (Lv et al., 2014) and gray matter thickness (Lin et al., 2017) early after RT, with the postcentral gyrus belonging to the frontoparietal network, the superior parietal lobule belonging to the CEN and the frontoparietal network, and the CEN being associated with working memory and executive and motor functions (Chen Q. et al., 2020). Our study showed that functional connectivity of PoCG.R was positively correlated with DSST, DST-FORWARD and DST-BACKWARD scores in PT3 group, suggesting that functional impairment in this brain region caused a decrease in executive function and memory in late delayed period, consistent with previous studies.

Both the anterior cingulate gyrus and lingual gyrus belong to the limbic system, which is very sensitive to radiation. Previous studies found significantly reduced (Qiu et al., 2018) or abnormal functional connectivity in the anterior cingulate gyrus after RT (Ma et al., 2016), and our study also found significantly reduced functional connectivity in the anterior cingulate gyrus. The lingual gyrus is located in the medial temporal lobe (MTL), which is a common target of RBI, and it is associated with visual memory encoding (Fulford et al., 2018) and working memory (Mattfeld et al., 2016). Our study found that the functional connectivity of the lingual gyrus was significantly reduced in the PT2 and PT3 groups, in which the functional connectivity of the lingual gyrus was positively correlated with AVLT recognition scores in PT2 group, which was consistent with the findings of previous studies. In addition, both the cuneus and middle occipital gyrus are involved in visual processing (Hahn et al., 2006), and their reduced functional connectivity suggests that RT leads to impaired visual memory in patients with NPC after RT (Cheung et al., 2003). The cuneus is also involved in cognitive control (Crockford et al., 2005), and its altered functional connectivity might suggest impaired cognitive control in patients with NPC after RT. Our study found that the functional connectivity of the left cuneus (CUN.L) to the HES.L and that of the left middle occipital gyrus (MOG.L) to the HES.L was significantly reduced after RT and was positively correlated with DST-BACKWARD and AVLT recognition scores. We speculate that RT may impair visual memory, cognitive control, and so on by impairing the function of these brain regions. The abnormal functional connectivity in these brain regions that are located away from the radiation field implies that RBI is not only limited to the exposed areas, and that other sensitive brain regions should also be evaluated closely. These findings suggest that altered functional connectivity patterns may be a potential biomarker of RBI in patients with NPC after RT.

### Dynamic Network Clustering Analysis—The Frequency of Various States in Each Subject F

Large-scale brain dynamics are characterized by repeating spatiotemporal connectivity patterns that reflect a range of putatively different brain states that underlie the dynamic repertoire of brain functions (Pedersen et al., 2018). Dynamic brain network analysis focuses on the spatiotemporal complexity and state transitions of brain networks, explores the rich dynamical complexity, and complements static brain network analysis to obtain more information (Stevner et al., 2019). This study is the first to explore the state transitions of RBI dynamic brain networks, and the results showed that the interaction effect of group and state is significant. In terms of group, the probability of the appearance of state 1 in the PT2 group was significantly greater than that of state 2. In terms of state, the probability of the appearance of PT2 group on state 1 was significantly greater than that of PT3 group, and the probability of the appearance of PT2 group on state 2 was significantly less than that of PT3 group. Brain network connectivity was lower in state 1 and higher in state 2. The significantly higher number of states with reduced brain network connectivity in the PT2 group implies that the brain functional impairment was more severe in the 6–12 months after RT than in the other groups, followed by more states with increased brain network connectivity in the PT3 group; this finding suggests that the functional brain network connectivity may have been gradually repaired or new functional connections were generated after 12 months post-RT, enabling the reduction of functional brain damage. Our previous study also found that RBI gradually worsened within 1 year and recovered after 1 year, and the present results are consistent with previous findings (Wang et al., 2012). Previous studies also suggest that RBI is a widespread and dynamic injury (Duan et al., 2016); the present study further confirms that RBI is closely associated with dynamic brain dysfunction and that RT significantly impairs functional connectivity throughout the brain. This study provides the first analysis of dynamic network indicators of RBI and reveals a higher complexity of brain activity, which is important to complement the interpretation of static network results and to uncover the pathogenesis of RBI.

### Strengths and Limitations

The current study features several strengths. First, to the best of our knowledge, this is the first study to examine the topological properties of static and dynamic functional networks in the brains of patients after RT for NPC, and it is the first study to perform a clustering analysis of dynamic functional networks. We obtained some functional imaging markers that could help in the early monitoring of RBI, including global properties (e.g., assortativity, hierarchy, network efficiency), nodal properties (nodal clustering coefficient), functional connectivity of static brain functional networks, and small-world variance, the frequency F of occurrence of various states of dynamic functional networks. Second, this is the first study to comprehensively explore the changes in cognitive function in patients after RT for NPC using a combination of multiple scales, remedying the deficiency of previous studies that only used the MoCA scale and thus were not sensitive enough. This study also comprehensively revealed the correlation between abnormal topological properties of brain functional networks and multiple cognitive impairments after RT, providing a basis for elucidating the pathogenesis of cognitive impairment after RT for NPC.

We acknowledge that the current study has some limitations. First, the sample size was small, especially the number of people in the 6–12 months after RT group, resulting in some indicators not reaching the level of significant differences. In the next study, we will continue to expand the sample size and work to validate the results of the present study. Second, our study was a cross-sectional study, which could not completely reveal the exact pattern of changes in brain function before and after RT. In the future, we will evaluate a cohort study of subjects to obtain the exact pattern of changes in brain function in RBI and the causal relationship between changes in brain network properties and radiation. Third, almost all subjects in this study were treated with chemotherapy and RT simultaneously, and it is possible that there is an effect of chemotherapy or a synergistic effect of chemotherapy and RT; therefore, future studies should include both chemotherapy-only and RT-only groups to assess this effect.

## Conclusion

For the first time, we synthesized the topological properties of static and dynamic functional networks of the brain, providing new insights into radiation-induced functional impairment after RT for NPC. The disruption of static and dynamic functional network stability, reduced network efficiency and reduced functional connectivity may be potential biomarkers of RBI. This study helps to further elucidate the pathogenesis of RBI and highlights the need for early monitoring of its occurrence, in which the small-world properties showed a tendency to be abnormal in static networks and small-world variance differed significantly in dynamic networks, which deserve attention.

## Data Availability Statement

The raw data supporting the conclusions of this article will be made available by the authors, without undue reservation.

## Ethics Statement

The studies involving human participants were reviewed and approved by the Ethics Committee of First Affiliated Hospital of Guangzhou University of Chinese Medicine. Written informed consent to participate in this study was provided by the participants’ legal guardian/next of kin.

## Author Contributions

XL, CQ, and SQ contributed to conception and design of the study. XL, CQ, HL, ML, and KZ organized the data. XL and CQ performed the data analysis and drafted the manuscript. All authors revised the manuscript and read and approved the submitted version.

## Conflict of Interest

The authors declare that the research was conducted in the absence of any commercial or financial relationships that could be construed as a potential conflict of interest.

## Footnotes

1 http://www.fil.ion.ucl.ac.uk/spm

2 http://www.nitrc.org/projects/gretna/
